# Gunshot injury to the left side of the neck with the lodged bullet in the carotid sheath: A case report

**DOI:** 10.1097/MD.0000000000041446

**Published:** 2025-02-14

**Authors:** Fatemeh Jahanshahi, Ali Saberi

**Affiliations:** a Student Research Committee, Faculty of Medicine, Iran University of Medical Sciences, Tehran, Iran; b Department of General Surgery, School of Medicine, Rasool Akram Medical Complex, Iran University of Medical Sciences, Tehran, Iran.

**Keywords:** bullet, case report, gunshot, neck injury, penetrating neck injury, retained bullet, Zone 2

## Abstract

**Rationale:**

Penetrating neck trauma involving the carotid artery is rare but highly lethal, requiring prompt and accurate diagnosis followed by immediate surgical intervention.

**Patient concerns:**

A 20-year-old male presented with a high-velocity gunshot wound to the left side of the neck. Despite the severity of the injury, the patient was asymptomatic and hemodynamically stable.

**Diagnose:**

Initial evaluation, including ultrasound imaging, revealed a bullet lodged within the left common carotid artery.

**Interventions:**

Due to the high risk of vascular complications, the patient underwent emergency surgery. The damaged artery segment was resected, and a primary end-to-end anastomosis was performed.

**Outcomes:**

The patient recovered well and was discharged without neurological deficits.

**Lessons:**

Early diagnosis and intervention are crucial in carotid artery injuries. This case demonstrates the limitations of computed tomography angiography in the presence of metallic artifacts and highlights the effectiveness of ultrasound for accurate diagnosis. Although penetrating neck injury management protocols typically recommend conservative management for asymptomatic Zone 2 injuries, surgical intervention is necessary due to the high-risk features of similar cases when the foreign object remains inside, and there is a risk of movement and severe damage. Adaptation of protocols based on individual injury characteristics can enhance patient outcomes. Although this is a rare case, similar cases have been reported, and it highlights the need to update the penetrating neck injury management protocols for the management of similar cases.

## 1. Introduction

Penetrating neck trauma presents a complex and challenging clinical scenario, particularly when major vascular structures such as the carotid artery are involved. Although arterial injuries constitute approximately 25% of all penetrating neck trauma cases, carotid artery involvement is seen in approximately 80% and vertebral artery in 43%.^[1]^

Mortality rates for major vascular injuries can reach up to 50%.^[2]^

Penetrating neck injuries are managed by dividing the neck into 3 anatomical zones:

Zone 1: from the clavicles and sternal notch to the cricoid cartilage. Since up to one-third of injuries may be asymptomatic initially, vascular and esophageal evaluations are recommended.
Zone 2: from the cricoid cartilage to the angle of the mandible. This area allows easier surgical access. Symptomatic injuries should undergo exploration. Asymptomatic injuries may be managed with mandatory exploration or directed evaluation. Angiograms are indicated for persistent hemorrhage, neurological deficits, Horner syndrome, or hoarseness.
Zone 3: from the mandible’s angle to the skull’s base. Injuries here can affect major blood vessels and cranial nerves and may be asymptomatic initially. Surgical access is challenging, but many vascular injuries can be treated by interventional radiology during the diagnostic angiogram.^[3]^

Management of carotid artery injuries requires prompt recognition and intervention to prevent life-threatening complications. Patients presenting with “hard signs” of vascular injury, such as external or internal hemorrhage, pulsatile hematoma, acute neurological deficits, or tracheal deviation, necessitate immediate surgical intervention. For hemodynamically stable patients without overt signs of vascular injury, diagnostic imaging modalities such as computed tomography (CT) arteriography, duplex ultrasonography, or color flow duplex are typically employed to delineate the extent of injury.^[4,5]^

This case report presents the emergency surgical management of a high-velocity gunshot injury to the left common carotid artery in Zone 2 of the neck with a retained bullet.

Despite the injury’s severity, the patient remained asymptomatic upon presentation, prompting a detailed evaluation using ultrasound imaging. The decision to proceed with emergency surgical exploration and repair was made due to the high risk of adverse vascular events associated with the presence of the bullet fragment within the carotid artery. The successful surgical intervention and uneventful postoperative recovery underscore the importance of timely and appropriate management in optimizing patient outcomes.

## 2. Case presentation

A 20-year-old male presented to our emergency department after being shot by a high-velocity bullet from an air rifle at a distance of about 6 meters. The bullet entered the left side of his neck midway through the sternocleidomastoid muscle and did not exit. The patient had no significant past medical or surgical history. On admission, his vital signs were stable, with a blood pressure of 108/76 mm Hg, a pulse of 84 beats/min, a respiratory rate of 18 breaths per minute, a temperature of 37 °C, and an oxygen saturation of 98%.

Upon examination 36 hours after the injury, the patient was fully conscious and alert with a Glasgow Coma Scale (GCS) of 15 and showed no signs of blood loss. Systemic examination revealed no abnormalities. The neurological assessment indicated normal pupil responses to light, unremarkable cranial nerve evaluation, normal reflexes, and power in all 4 limbs. A digital rectal examination revealed a normal tone. During the physical examination, a bullet entry wound sized 0.5 × 0.5 cm without any bruise and hematoma was observed, but there was no corresponding exit wound.

Laboratory tests showed a white blood cell count of 9.17 × 10^3^/mm³, a hemoglobin level of 11.8 g/dL, hematocrit of 34.9%, platelet count of 221 × 10^3^/μL, activated partial thromboplastin time of 28.2 seconds, an international normalized ratio of 1.37, creatinine level of 0.74 mg/dL, urea level of 14 mg/dL, aspartate aminotransferase level of 28 U/L, alanine aminotransferase level of 10 U/L, sodium level of 142 mEq/L, and potassium level of 3.7 mEq/L.

A grayscale ultrasound examination revealed that both sides’ common, internal, and external carotid arteries had normal diameters. Color Doppler imaging showed normal arterial flow with low resistance in the common and internal carotid arteries, with no signs of significant stenosis that could cause hemodynamic disturbances. The blood flow in the vertebral arteries was in the same direction as the carotid artery.

An echogenic foreign body measuring 5 to 6 mm was observed in the posterior part of the middle segment of the left common carotid artery, corresponding to the bullet entry point. The bullet had passed through the posterior wall of the common carotid artery and penetrated 3 mm into the lumen of the artery. There were no signs of extensive hematoma at the bullet entry point into the carotid artery, and it appeared that the bullet sealed the entry site. Despite aliasing in the arterial flow in this area, there were no signs of increased peak systolic velocity or stenosis and no evidence of internal lumen flap formation indicative of dissection.

The patient was diagnosed with a common carotid artery injury and was promptly prepared for surgery. He received 1500 IU of tetanus antitoxin intramuscularly and 2 g of cefazolin intravenously. Under general anesthesia, an oblique incision was made along the anterior border of the left sternocleidomastoid muscle to expose the common carotid artery. Before applying vascular clamps, 5000 units of heparin were administered intravenously. The internal and external carotid arteries were clamped distal to the gunshot injury area. The high-velocity gunshot had traversed the common carotid artery near the bifurcation.

**Figure 1.**
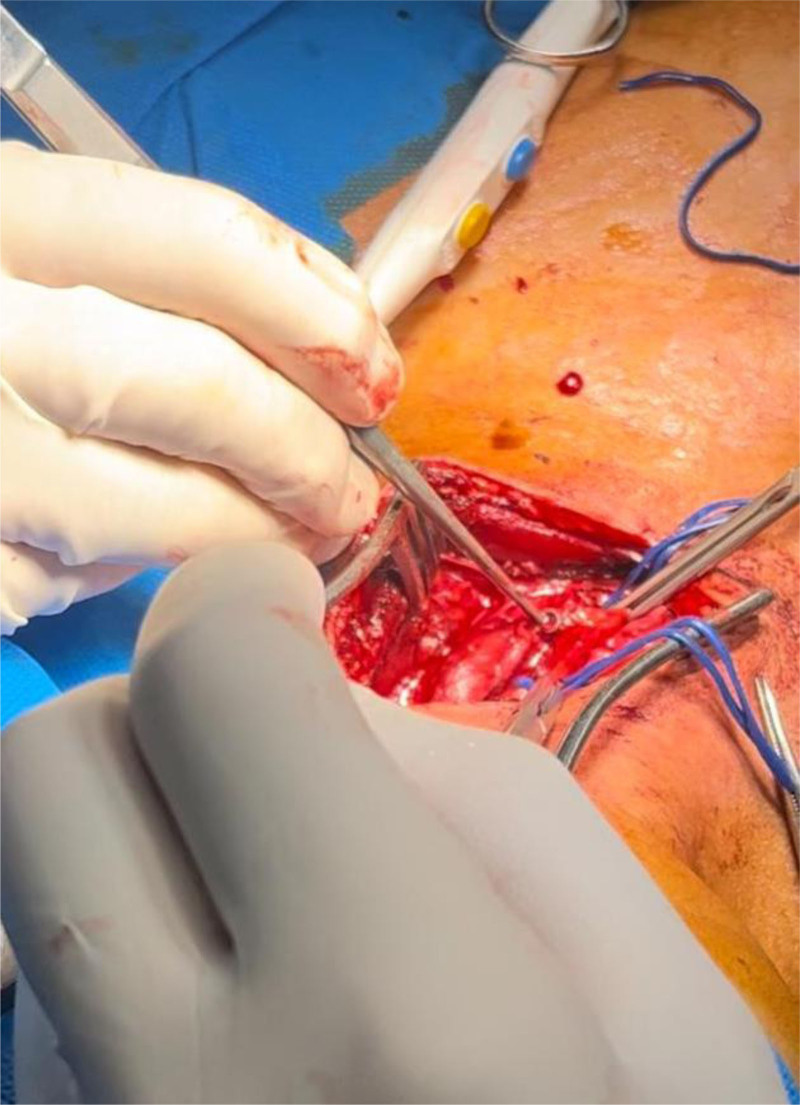
Intraoperative image of the bullet removal from the carotid artery.

The damaged segment of the common carotid artery was resected proximally and distally, and primary end-to-end anastomosis was performed using continuous 6/0 Prolene sutures. The wound was irrigated with saline containing rifampicin, and a drainage tube was placed before closing the wound (Fig. 1) (Video S1, Supplemental Digital Content, http://links.lww.com/MD/O398).

Postoperatively, the patient exhibited no neurological deficits. He was discharged on the 8th day after surgery with a prescription for aspirin 80 mg daily. There were no complications during his hospital stay or upon discharge.

## 3. Discussion

Penetrating neck trauma, particularly gunshot wounds, poses significant risks due to the density of vital structures in the neck, with mortality rates reaching up to 50% for major vascular injuries.^[2,6]^

Studies have shown that injuries to the head and neck from firearms result in a substantial health and financial burden, with a high incidence of 20.1 cases per 100,000 hospitalizations and a case fatality rate of 32.4% for intentional injuries.^[7]^

Specialized surgical care for patients with neck injuries, especially those involving hollow neck organs, is crucial for successful treatment and reducing lethal outcomes, particularly in military settings with new types of weapons.^[6]^ Additionally, the removal of retained foreign bodies in the neck, such as from firearm injuries, is rare but can be successfully managed through surgical intervention, ensuring patient recovery and resumption of normal activities.^[8]^

Due to the high prevalence and critical features of penetrating neck injuries, numerous guidelines have been developed for the management of these injuries. However, none of these guidelines address the management of stable cases with remaining foreign bodies, such as bullets in the neck.

In penetrating neck injury management protocol, Zone 2 refers to the mid-neck region between the cricoid cartilage and the angle of the mandible. According to this protocol, asymptomatic patients with injuries in Zone 2 are often managed conservatively with careful observation, imaging studies, and selective surgical exploration if deemed necessary based on the presence of certain risk factors.

In the presented case, the patient sustained a high-velocity gunshot injury to the left carotid artery within Zone 2 of the neck. Despite the potentially severe nature of the injury, the patient remained asymptomatic upon presentation, exhibiting stable vital signs and no overt signs of neurological deficits. As per the mentioned protocol, asymptomatic patients in Zone 2 typically undergo a thorough evaluation, including physical examination, laboratory tests, and imaging studies, to assess the extent of the injury and potential vascular involvement.

The initial evaluation of the patient involved a comprehensive neurological examination, laboratory investigations, and ultrasound imaging, which revealed the presence of a foreign body consistent with the gunshot projectile lodged within the left common carotid artery. Despite the absence of immediate clinical manifestations, the presence of a foreign body within a major vascular structure necessitated prompt intervention to mitigate the risk of catastrophic complications such as thrombosis, embolism, or hemorrhage.

Although this protocol generally advocates for conservative management of asymptomatic Zone 2 injuries, certain high-risk features, such as foreign body penetration of a major artery, may warrant deviation from the standard approach. In this case, the decision was made to proceed with emergency surgical exploration and intervention due to the high likelihood of adverse vascular events associated with the presence of the bullet fragment within the carotid artery.

Although this case is extremely rare, other similar cases have also been reported, and it is recommended that the management of these cases be incorporated into the management guidelines.

In 2023, Summer Hassan et al documented the case of an 18-year-old Caucasian male admitted with a neck injury from an air rifle. On examination, he presented with active bleeding and a moderate hematoma causing tracheal displacement, yet remained stable with a GCS of 15 and no bruit.

The entry wound, located on the right of the neck’s midline, lacked an exit wound but exhibited minor bleeding, subcutaneous emphysema, and a moderate hematoma. A contrast-enhanced computed tomography angiogram revealed a 6.57 mm metal pellet lodged 1.2 cm behind the mid-right common carotid artery and 3.5 cm proximal to the carotid bifurcation near the C6 vertebra.

Treatment involved neck exploration under general anesthesia, repair of the common carotid artery, bullet extraction, and wound irrigation. The patient was discharged 2 days post-surgery without complications. This case underscores the potential risks associated with retained small airgun pellets, including embolization and vascular injury. Surgical exploration of the neck remains the preferred approach for managing penetrating carotid injuries in stable patients with retained foreign bodies. Preoperative planning, aided by imaging such as CT angiography, is essential for guiding surgical intervention effectively.^[9]^

Rehan Muhammad et al, 2023, reported a 41-year-old male presented to the emergency department with a right-side penetrating neck injury from a metallic fragment. He had a heart rate of 107 beats/min, blood pressure of 135/87 mm Hg, respiratory rate of 18 breaths/min, oxygen saturation of 98%, and a GCS score of 15/15. Examination showed a stable patient with an entry wound in Zone 1 of the neck and no exit wound. A CT scan revealed a 1.5 cm metallic fragment between the internal jugular vein and the common carotid artery.

The patient underwent surgery the same day. A right horizontal incision was made, and the shrapnel was removed without causing further injury. Postoperatively, he remained stable and was discharged the next day with a follow-up appointment in 3 weeks.^[10]^

In 2020, Cesar Reategui reported on a 31-year-old Caucasian male who presented to the emergency room after accidentally impaling his right neck with a metallic fragment while hammering metal at work. With no significant medical history, the patient showed no signs of distress and talked comfortably, although he had some discomfort in the area. Physical examination revealed a 1 cm wound in Zone 2 of the neck with no hematoma or active bleeding. His vital signs were stable, and laboratory results were within normal limits.

A CT angiography revealed a 9 mm metallic fragment embedded in the right sternocleidomastoid muscle, 7.4 mm anterolateral to the right common carotid artery, without vascular injury. The patient underwent surgery for foreign body removal under fluoroscopic guidance, fiberoptic esophagoscopy, and bronchoscopy.

In conclusion, Reategui claimed that the management of lodged foreign bodies in the neck, as demonstrated in this case, should be included in future guidelines for penetrating neck trauma. Current guidelines do not adequately address the management of retained foreign bodies in asymptomatic patients with such injuries. Detailed imaging, precise localization, and careful surgical planning are crucial for safely and effectively removing foreign bodies near vital structures. Incorporating these strategies into guidelines will help standardize care and improve outcomes for patients with similar injuries.^[11]^

In 2014, Peter A. Ongom et al reported on a 27-year-old African–Ugandan woman of Nilotic ethnicity who was referred to a tertiary hospital in Uganda after sustaining complex injuries from an inadvertent AK-47 rifle gunshot. The gunshot caused a large, ragged entry wound on the right side of her face with no exit wound. Initial care and radiological imaging revealed a comminuted fracture of her mandible and a bullet lodged in her neck, anterior to the 6th and 7th cervical vertebrae.

Standard wound debridement was performed, and a CT scan later showed the bullet had shifted cephalad, lying partially anterior to the 5th cervical vertebra and within her carotid sheath. The patient also suffered injuries to her facial and trigeminal nerves, as well as her middle ear. The “wandering” bullet was successfully removed surgically without causing further damage to her neck structures.

In conclusion, Ongom et al demonstrated that thorough imaging and careful surgical intervention are crucial in managing complex gunshot injuries involving migrating foreign bodies, ensuring no further damage to vital structures.^[12]^

In 1992, J. W. Nicol reported on a 33-year-old man who had a foreign body in his right pharyngeal region from a gunshot wound sustained in the USA. The injury caused damage to his upper lip, hard and soft palates, oropharynx, and mid-facial skeleton. Initially stable, the patient had hyponasal speech, mild dysphagia, and scars from the injury, with a small hard mass in the right jugulodigastric region. Radiographs showed a right-sided opacity near the third cervical vertebra.

Six months later, he developed neck pain, dysphonia, and increased dysphagia. Indirect laryngoscopy and radiographs revealed the foreign body protruding into the pyriform fossa, causing edema. Surgery was performed, involving anesthesia, a tracheostomy, and the removal of a large bullet fragment from the right supraglottic area. The patient recovered with normal airway, voice, and swallowing.

Nicol concluded that patients with stable conditions and no neurological deficit, despite bullet fragments near vital structures, pose a management challenge. He recommended the removal of retained bullet fragments to prevent complications.^[13]^

CT angiography offers a noninvasive, quick, and dependable alternative to arteriography, delivering crucial diagnostic insights into vascular and aerodigestive injuries and bullet paths. It is a commonly used diagnostic tool for evaluating vascular injuries.^[9]^

However, in this case, the presence of a bullet within the artery wall would likely cause significant artifacts in the imaging, rendering CT angiography less effective for accurate diagnosis. Artifacts from metallic foreign bodies can obscure important anatomical details and impede the visualization of vascular structures, making it difficult to assess the extent of injury and plan appropriate surgical interventions.

According to the limitations of CT angiography in the presence of metallic artifacts, ultrasound imaging presents a viable alternative for diagnosing vascular injuries. Ultrasound offers several advantages, including real-time visualization of blood flow, the absence of ionizing radiation, and the ability to detect foreign bodies and their relationship to vascular structures. In this case, ultrasound was instrumental in identifying the foreign body lodged within the left common carotid artery and assessing the extent of vascular involvement without the confounding effects of imaging artifacts. However, in cases involving high-velocity gunshot injuries, the presence of metallic foreign bodies within the vascular structures can pose significant diagnostic challenges. CT angiography, while commonly used, may be less effective due to artifacts caused by the metallic presence, obscuring critical anatomical details and complicating the assessment of vascular injuries. In such scenarios, alternative imaging modalities like ultrasound offer distinct advantages.

The surgical approach involved careful dissection and repair of the injured carotid artery segment, including resection of the damaged tissue and primary end-to-end anastomosis. Postoperatively, the patient demonstrated an uneventful recovery without neurological deficits, highlighting the efficacy of timely surgical intervention in preventing catastrophic complications associated with vascular injuries in Zone 2 of the neck.

While neck injury guidelines provide valuable guidelines for managing penetrating neck injuries, clinicians must exercise clinical judgment and adapt their approach based on individual patient characteristics and injury patterns. In cases involving high-risk features, such as foreign body penetration of major vessels, prompt surgical intervention may be warranted to optimize patient outcomes and prevent potentially life-threatening complications. The head and neck are small areas with many important structures close together, meaning that even a slight movement of a lodged bullet can cause serious damage to nerves, blood vessels, bones, and other tissues. However, surgical exploration of a bullet in these areas carries risks, including harm to surrounding tissues, infections, severe hemorrhage, and the potential for the bullet to move during or after the procedure. While surgical exploration may be necessary, these risks must be carefully weighed in each case to ensure the best possible outcome for the patient.^[12,14]^

## 4. Limitation

This study has several limitations. As a rare case, there is limited documentation of similar instances in the literature. Consequently, there is insufficient information on this topic, and current guidelines primarily recommend conservative management for such cases.

## 5. Conclusion

In conclude, although the existing guidelines recommend that asymptomatic patients with penetrating neck trauma in Zone 2 be managed conservatively, the presence of a retained foreign body in this area poses a risk of foreign body migration and injury to vital organs. Therefore, it is recommended that in similar cases with an entrance wound but no corresponding exit wound, the foreign body should be removed promptly due to the critical nature of the condition. Despite the rarity of such similar cases, it would be beneficial to include them in the penetrating neck trauma management guidelines.

## Author contributions

**Conceptualization:** Ali Saberi, Fatemeh Jahanshahi.

**Data curation:** Fatemeh Jahanshahi.

**Investigation:** Fatemeh Jahanshahi.

**Supervision:** Ali Saberi.

**Writing – original draft:** Fatemeh Jahanshahi.

**Writing – review & editing:** Fatemeh Jahanshahi, Ali Saberi.

## Supplementary Material


